# Necessity of immediate cardiopulmonary resuscitation in trauma emergency

**DOI:** 10.1186/1749-7922-5-25

**Published:** 2010-08-25

**Authors:** Baitello Andre Luciano, Gonzáles Ferreira Marcela, Espada Paulo Cesar, José Maria Pereira de Godoy

**Affiliations:** 1 Department of Trauma in Medicine School of São Jose do Rio Preto-FAMERP-Brazil; 2 Department of Cardiology and Cardiovascular Surgery in Medicine School in São José do Rio Preto (FAMERP) and CNPq (National Council for Research and Development)-Brazil

## Abstract

The ability to respond quickly and effectively to a cardiac arrest situation rests on nurses being competent in the emergency life-saving procedure of cardiopulmonary resuscitation. The objective of the current study was to evaluate the types of trauma and survival of patients that require immediate cardiopulmonary resuscitation in trauma emergencies. A total of 13301 patients treated as accident victims between July 2004 and December 2006 were evaluated in a prospective study. Patients requiring immediate cardiopulmonary resuscitation at admission were identified. The type of injury and the survival of these patients were evaluated.

Of the 65 patients included in the study, 30% had suffered from gunshot wounds, 19% had been run over, 18% had been involved in car crashes, 13% in motor cycle accidents, 9% stabbings, 1% by cycle accidents and 10% other types of accidents including burns, hangings and falls. In only 12 of these patients, immediate resuscitation was successful and procedure such as chest drainage, exploratory laparotomy and interventions in the surgical center were performed. However all patients evolved to death; eight within 24 hours, two between 24 and 48 hours and the other 2 after 48 hours.

Immediate cardiopulmonary resuscitation after accidents is a sign of high mortality requiring further studies to review indication and the ethical aspects involved.

## Introduction

In the medical practice, the different scenarios in which cardiorespiratory resuscitation (CPR) may be applied must be taken into account. CPR is crucial in patients that arrive in emergency rooms or suffer a cardiac arrest in public places or in their homes. It is also critical in hospitalized patients with potentially reversible diseases, who suffer cardiac arrest as an unexpected event during their evolution [1].

The latest guidelines for CPR and emergency cardiovascular care published by the American Heart Association include substantial changes to the algorithms for basic life support and advanced cardiovascular life support [2].

The most critical emergency situation seen in cardiac surgical units is the need for chest reopening. While senior nurses often manage cardiac arrest they currently are not trained to open chests, which can be a life-saving action if performed efficiently [3]. The ability to respond quickly and effectively to a cardiac arrest situation rests on nurses being competent in the emergency life-saving procedure of CPR. The study findings present strong evidence to support the critical role of CPR training in ensuring that nursing student's progress to competent and confident responders in the event of a cardiac related emergency [4].

The objective of the current study was to evaluate the types of injuries and the survival of patients who require immediate cardiopulmonary resuscitation in trauma emergencies.

## Method

A total of 13301 accident victims treated in the accident and emergency department of Hospital de Base in São José do Rio Preto between July 2004 and December 2006 were evaluated in a prospective study. Patients requiring immediate cardiovascular resuscitation on admission were identified. The types of injury and survival of these patients were evaluated.

This study was approved by the Research Ethics Committee.

## Results

Sixty-five patients arrived in the Accident and Emergency Department with an arterial blood pressure of 0/0 mmHg. Table 1 shows the main types of injuries.

**Table 1:** Frequency of the types of injuries in these patients

Injury	N
Gunshot wounds	20
Stabbings	4
Car crashes	12
Motor cycle accidents	9
Run over	12
Bicycle accidents	1
Overturned car	1
Hangings	1
Severe burns	1
Falls	2
Others	2

In only 12 of these patients, immediate resuscitation was successful and subsequent procedures such as chest drainage, exploratory laparotomy and interventions in the surgical center were performed, but not had improvement in the neurological. The specific kinds of trauma in each patient were not identified. Even so all the patients evolved to death; eight died within 24 hours, two between 24 to 48 hours and the other two after 48 hours.

## Discussion

The current study shows that immediate cardiopulmonary resuscitation is a factor for high mortality in victims of trauma emergencies. The few published studies on this subject confirm this high mortality rate [5,6]. Instead of insisting on aggressive measures to resuscitate trauma patients in extremis on presentation, the authors suggest we should redirect that fervor toward efforts made to promote trauma awareness and injury prevention programs [6]. Another aspect to be evaluated is the cost of these interventions for patients who have a low probability to survive. Studies show that the duration of cardiopulmonary resuscitation was positively associated with the elevation of cardiac markers [7]. Study related that we cannot decide to give up and terminate resuscitation in any cardiopulmonary arrest on arrival due to penetrating trauma patients and cannot define salvageable patients. However, our data show that 30-min resuscitation is thought to be relevant and that we should not give up on resuscitation because of the time interval without return of spontaneous circulation after arrival at the hospital [8].

Another factor to be discussed is related to ethics and organ donations that these patients may provide, as, in the current study donations of organs occurred in only one case. On the other hand in teaching hospitals, the academic importance should be considered in the treatment of these patients. The prolongation of the life of patients with high risk of mortality only causes more suffering to relatives in respect to the expectations of the prognosis of the patient.

In the context of a community-wide focus on resuscitation, the sequential implementation of 2005 American Heart Association guidelines for compressions, ventilations, and induced hypothermia significantly improved survival after cardiac arrest. Further study is required to clarify the relative contribution of each intervention to improved survival outcomes [9].

## Conclusion

Immediate cardiopulmonary resuscitation in accident victims is a sign of high mortality rates. Further studies are necessary to review indications and ethical aspects.

## Competing interests

The authors declare that they have no competing interests (political, personal, religious, ideological, academic, intellectual, commercial or any other) in relation to this manuscript.

## Authors' contributions

BAL participated and contributed to all phases of the study. FMG participated and contributed to all phases of the study. EPC participated and contributed to all phases of the study. GJMP participated and contributed to all phases of the study. All authors read and approved the final manuscript.
